# TREM2 Mediates Microglial Anti-Inflammatory Activations in Alzheimer’s Disease: Lessons Learned from Transcriptomics

**DOI:** 10.3390/cells10020321

**Published:** 2021-02-04

**Authors:** Feng Xue, Heng Du

**Affiliations:** 1 Department of Pharmacology & Toxicology, School of Pharmacy, The University of Kansas, Lawrence, KS 66045, USA; feng.xue@ku.edu; 2 Higuchi Biosciences Center, The University of Kansas, Lawrence, KS 66045, USA

**Keywords:** Alzheimer’s disease, microglia, transcriptomics, TREM2/Trem2, neuroinflammation

## Abstract

Alzheimer’s disease (AD) is a lethal neurodegenerative disorder primarily affecting the aged population. The etiopathogenesis of AD, especially that of the sporadic type, remains elusive. The triggering receptor expressed on myeloid cells 2 (TREM2), a member of TREM immunoglobulin superfamily, plays a critical role in microglial physiology. Missense mutations in human *TREM2* are determined as genetic risk factors associated with the development of sporadic AD. However, the roles of TREM2 in the pathogenesis of AD are still to be established. In this review, we outlined the influence of Trem2 on balance of pro- and anti-inflammatory microglial activations from a perspective of AD mouse model transcriptomics. On this basis, we further speculated the roles of TREM2 in different stages of AD, which may shed light to the development of TREM2-targeted strategy for the prevention and treatment of this neurodegenerative disorder.

## 1. Introduction

Alzheimer’s disease (AD) is a heterogeneous and chronic neurological disorder in which progressive cognitive decline and gradual neurodegeneration converge [1,2]. Consistent with the histopathological characteristics of AD brains, amyloid beta (Aβ) and hyperphosphorylated Tau are well accepted mediators of synaptic failure and neuronal death in this neurodegenerative disorder [1,2]. Mutations in genes encoding amyloid precursor protein (APP), presenilin 1 (PS1) and presenilin 2 (PS2) that promote amyloidosis and/or tauopathy are closely associated with the development of familial AD (FAD) [3,4]. However, the etiopathogenesis of sporadic AD (SAD) remains elusive. Alongside Aβ plaque and neurofibrillary tangles (NFTs), the progressively developed chronic neuroinflammation is an important feature in FAD and SAD pathogenesis [5,6,7,8]. Microglia are brain-resident myeloid cells that play major roles in the generation and propagation of neuroinflammation in health and disease [9,10,11]. In recent years, analysis of AD genetic susceptibility loci through genome-wide association studies (GWAS) has identified a variety of variants clustering in immune response [12,13,14,15,16]. Moreover, transcriptomics studies suggest the relationship between several AD risk loci and microglial immune response to Aβ toxicity [17,18]. These findings support a hypothesis that microglia contribute to AD pathogenesis.

**Figure 1:**
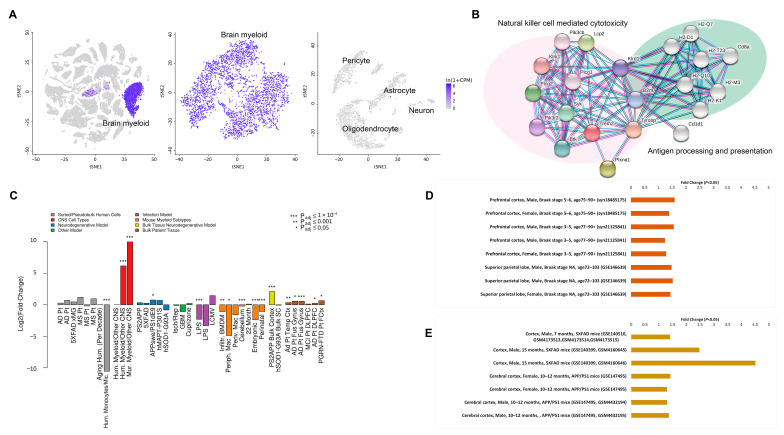
*Trem2*/*TREM2* expression patterns in Alzheimer’s disease (AD) mouse models and AD postmortem samples. (**A**) *Trem2* gene expression in 3-month-old *Mus musculus* C57BL/6 brain myeloid cells. The scRNAseq data were acquired from Tabula Muris [30]. (**B**) Protein interaction network of Trem2 in *Mus musculus* innate immunity. The self-learning interaction network was generated by STRING [31]. (**C**) *Trem2*/*TREM2* upregulations in multiple AD animal models and AD postmortem samples. Metadata were acquired from The Myeloid Landscape 2 [44]. (**D**) *TREM2* upregulations in AD postmortem samples (*p* < 0.05). The snRNAseq gene expression data were acquired from scREAD [45]. (**E**) *Trem2* upregulations in microglia of 5XFAD and APP/PS1 AD mouse models (*p* < 0.05). The snRNAseq gene expression data were acquired from scREAD [45].

The triggering receptor expressed on myeloid cells 2 (TREM2) is a member of TREM immunoglobulin superfamily [19]. Recent studies revealed relationships between TREM2 and human diseases. Severe neurological sequelae occur in patients with loss-of-function variants of *TREM2* [20]. Null mutations in *TREM2* is a cause of Nasu-Hakola disease (NHD) [21], a rare autosomal recessive brain disorder characterized by frontal lobe syndrome, early-onset dementia, and bone cysts [22]. Patients with heterozygous missense mutations in *TREM2* demonstrate increased susceptibility to chronic neurodegenerative disorders including frontotemporal dementia (FTD)-like syndrome [23], Parkinson’s disease (PD) [24], and AD [20,25,26,27]. Single-cell RNAseq (scRNAseq) and single-nucleus RNAseq (snRNAseq) transcriptomics on mouse models revealed that *Trem2* is mainly expressed by microglia in the central nervous system (CNS) (Figure 1A) [28,29,30]. Protein network analysis showed that Trem2 is co-enriched with proteins in innate immunity (Figure 1B) [31], implicating its relevance to microglial function and innate immune signaling. Indeed, emerging evidence suggests that Trem2 promotes microglial proliferation and survival, as well as regulating microglial phagocytosis and metabolism in the brain of mouse models [20]. In AD-related conditions, Trem2 has an intertwined relationship with lipoproteins, anionic lipids, and Aβ, which contributes to microglial metabolism remodeling, as well as promotes microglial phagocytosis of cell debris and Aβ [32,33]. Furthermore, Trem2 is proposed to play a critical role in inducing microglial anti-inflammatory activation in AD-related conditions [34,35]. These effects support a hypothesis that TREM2 is involved in the development of AD and is a potential target for the prevention and treatment of AD. However, the role of TREM2 in AD etiopathogenesis is complicated by the conflicting impacts of *Trem2* deficiency on brain amyloidopathy and tauopathy in multiple lines of mouse models [36,37,38,39,40]. Moreover, studies on the protective effects of *Trem2* overexpression have produced inconsistent results [41,42,43]. These disparities may be interpreted by a hypothesis that TREM2 plays a double-edged sword role in AD pathogenesis [34,42,43].

Microglia are the major innate immune cells in CNS and serve as tissue-resident myeloid phagocytes in infection and neurodegeneration. Recently, there is an increasing consensus that microglia play multifaceted roles in AD progression [46]. In AD-related conditions, they phagocytize Aβ and cell debris, respond to the change in homeostasis, and differentiate to be “activated microglia”. Microglial activation is a precisely regulated process, which includes immune signaling activations, morphological changes, surface marker changes, secretion of immune cytokines, and cellular proliferation. Both pro- and anti-inflammatory microglial activations further mediate chronic neuroinflammation leading to severe synaptic injury and neuronal lesions [8,10,45]. The pro-inflammatory microglial activations were well studied and summarized previously [47], while Trem2 mediates a novel form of anti-inflammatory microglial activation in AD mouse models. In this context, the influence of Trem2 on microglial anti-inflammatory activation has raised an intriguing question whether TREM2 contributes to the chronic neuroinflammation in human AD. In this review, we summarized current knowledge and discussed the role of Trem2 in the balance of microglial pro- and anti-inflammatory activations and its possible contributions in AD chronic neuroinflammation from a transcriptomics perspective.

## 2. Trem2 and Disease-Associated Microglia (DAM)

In multiple lines of AD mouse models, Trem2 is prominently upregulated both at transcription level and at translation level in AD-sensitive brain regions in a disease progression-related pattern (Figure 1C) [44,48,49,50,51]. TREM2 upregulation had also been determined in peripheral mononuclear cells from AD patients and mild cognitive impairment [52,53], implicating that the TREM2 upregulation may be a systemic response of myeloid cells with the progression of AD. The snRNAseq data on individual AD postmortem sample and AD mouse model also revealed that TREM2/Trem2 was upregulated in AD pathology and AD-related conditions (Figure 1D,E) [45,54,55,56,57]. In addition, Trem2 is also upregulated in an amyloidosis progression-related pattern, predominantly in plaque-surrounding microglia [58]. Consistent with such a spatiotemporal relationship between plaques and Trem2 expression, the interaction partners of Trem2 including Aβ [59,60], neutral, and anionic lipids [61], as well as Apolipoprotein E4 (APOE4) [52,62] and CD33 [63] are determined as inducing factors for Trem2 upregulation. In addition, epigenetic regulation of Trem2 has been noticed [48,64]. Moreover, Trem2 is sensitive to pro-inflammatory signaling, at least in an in vitro context. Anti-inflammatory cytokines such as interleukin-4 (IL-4) and IL-13 [65] can upregulate Trem2 expression. Whereas Trem2 is downregulated by NF-κB-mediated miRNA-34a [66], lipopolysaccharides (LPS)-induced pro-inflammatory signaling [67], as well as pro-inflammatory cytokines [68]. So far, the leading factors that induce Trem2 upregulation in AD-related conditions remain unclear. One possibility is that the components of Aβ plaques synergistically regulate Trem2. This is supported by a study from Neher’s group that Trem2 upregulation is triggered in microglia during their migration to plaques [69]. Another possibility is that Trem2 was induced by multiple factors of neuroinflammation, because it was a Stage 2 DAM marker and upregulated at the late stage of amyloidosis and neuroinflammation (Table 1) [9]. In view of the potential influence of Trem2 upregulation on Aβ clearance via microglial phagocytosis [41,42,43], the intersecting relationship between Trem2 and brain amyloidosis opens up an argument about the roles of enhanced TREM2 expression in the pathogenesis of AD.

**Figure 2:**
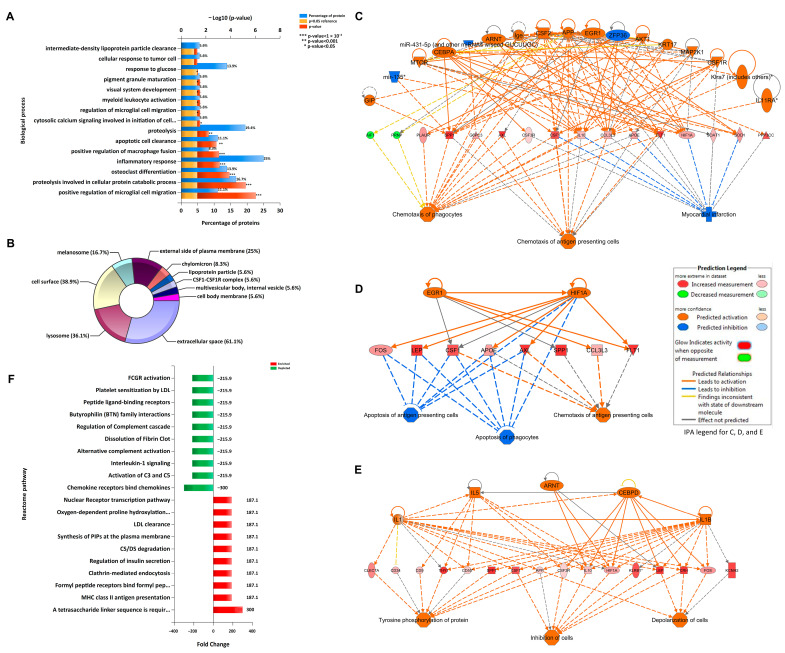
Pathway enrichments and regulation networks of Trem2-dependent DAM markers. (**A**) Gene ontology biological process pathway enrichment analysis of the major DAM markers. (**B**) Gene ontology cellular component enrichment analysis of the major DAM markers. (**C**) Upstream regulators and downstream chemotaxis effect of the DAM markers (*Spp1*, *Axl*, *Csf1*, and *Apoe*). (**D**) Upstream regulators and downstream anti-apoptosis effect of the DAM markers (*Csf1*, *Apoe*, *Axl*, and *Spp1*). (**E**) Upstream regulators and downstream cellular depolarization effect of the DAM markers (*Clec7a*, *Cd9*, *Spp1*, and *Csf1*). (**F**) Fold enrichment analysis of the neurodegeneration-related modules (DAM and DAM-related markers) against the LPS-related modules as background. Bioinformatics analysis methods were appended in “Supplementary File—Materials and Methods”. The data citation information used in the bioinformatics analysis is listed in “Supplementary File—Transcriptomics data list”.

With the concept borrowed from studies on macrophage, in vitro cultured microglia have been categorized into two subgroups, “M1-like” and “M2-like”. Microglia demonstrating pro-inflammatory activations are defined as the “M1-like”. While those demonstrating anti-inflammatory activations are defined as the “M2-like”. However, these subgroups were not correlated with the microglial phenotypes under in vivo pathological conditions. Recent scRNAseq and snRNAseq transcriptomics data highlight the heterogeneity of microglia in health and disease [71]. A predominant subgroup of microglia clustered as disease-associated microglia (DAM) were identified in 5XFAD mice and subsequently verified in multiple mouse models of AD and other neurodegenerative diseases [9,72]. The pathway enrichment analysis on DAM markers predicts multiple functions of the DAM subgroup. The gene ontology (GO) biological process enrichment analysis implicates the involvement of DAM in positive regulation of microglial cell migration, proteolysis involved in cellular protein catabolic process, osteoclast differentiation, and inflammatory response, as well as positive regulation of macrophage fusion (*p* < 0.001) (Figure 2A). Furthermore, the cellular component enrichment analysis suggests the distribution of DAM markers in the extracellular space, lysosome, cell surface, melanosome, and external side of plasma membrane (*p* < 0.001) (Figure 2B). Therefore, these DAM markers are proposed to be associated with the regulation of microglial chemotaxis, proteolysis, inflammatory response, and lysosome biosynthesis. Of note, Trem2 signaling is one of the critical upstream pathways of DAM in AD mouse models [72,73]. The DAM subgroup initiates in 1-month old 5XFAD mice, and *Trem2* knockout can block the subgrouping. Meanwhile, *Trem2* is a Stage 2 DAM upregulation marker, which suggested that Trem2 signaling activation upregulates *Trem2* itself in AD-related conditions. So far, the precise functions of the Trem2-dependent DAM and its significance in AD pathogenesis are not fully resolved yet. It is generally accepted that the DAM is protective in slowing down the progression of AD pathology, although recent studies further defined the deleterious “pro-inflammatory” and the protective “anti-inflammatory” sub-subgroups in the DAM subgroup [74,75]. Indeed, the summary of putative and verified upstream regulators of the major DAM markers revealed that both pro- and anti- inflammatory signaling pathways were involved in DAM formation (Table 1). It is possible that the Trem2-dependent DAM plays multiple roles in a sophisticated manner.

We further performed bioinformatics analysis on the major Trem2-dependent DAM markers. Regulator effect network analysis using QIAGEN Ingenuity Pathway Analysis (IPA) suggests that Csf1/Csf2 signaling alongside Tnf/Ifng-Hif1a/Stat1 and Tgfb1-Mapk-Apoe axes constitute major upstream regulation networks of the major DAM markers (Figure 2C–E and Table 1) [76]. The Csf1/Csf2 signaling is the key upstream pathways that regulates myeloid cell proliferation, development, and homeostasis [77]. The Tnf/Ifng axis is the major pro-inflammatory cytokine signaling in neuroinflammation. Tgfb1-Mapk-Apoe axis can maintain homeostasis of macroglia and restrain pro-inflammatory activation of microglia [78]. Thus, the upstream regulation network of Trem2-dependent DAM is a complex composed of both pro- and anti-inflammatory signaling pathways (Table 1). Additionally, the functions of Trem2-dependent DAM may include depolarization, differentiation, and chemotaxis of microglia [79,80]. The Trem2-dependent DAM subgroup was also defined as the neurodegeneration-related modules in hierarchical clustering analysis of scRNAseq data [81]. Meanwhile, the LPS-related modules were identified as the major pro-inflammatory activation of microglia in AD mouse models.

To explore the major role of Trem2-dependent DAM in neuroinflammation, we performed a fold enrichment analysis of the neurodegeneration-related modules against the LPS-related modules as background using FunRich3.1 [81,82]. The enrichment in innate immune pathways of the neurodegeneration-related modules included antigen presentation and endocytosis, while the depletion in pro-inflammatory activations included chemokines, complement system, and Il1 signaling (Figure 2F). This analysis added supportive evidence to the documented anti-inflammatory effects of Trem2 signaling [83,84]. However, the anti-inflammatory properties of Trem2-dependent DAM may be feeble because the genetic *Trem2* depletion shows less effect on pro-inflammatory gene expression in some AD mouse models [37,61]. In this regard, it is still unclear whether Trem2 modulation can influence pro- or anti-inflammatory microglial activations. In addition, such disparities may also remind us to pay attention to the dose effect of Trem2 as well as the interaction of Trem2 with other inflammatory pathways. Noteworthy, it should be mindful that mouse models may not comprehensively recapitulate microglial changes in AD patients based on the inconsistent microglial transcriptomics profiling between AD mouse models and AD [54,56,81,82]. Therefore, further patients-based studies may help to determine the roles of TREM2 in shaping microglial phenotypes and DAM functions in AD.

## 3. TREM2 and AD Pathology

Chronic neuroinflammation is an important feature in AD pathology. The double-edged sword roles of inflammation in the pathogenesis of AD have been intensively reviewed [8,45,83]. On the bright side, inflammation modulates microglial maturation and activation and promotes the clearance of cell debris and Aβ via microglial phagocytosis. On the dark side, neuroinflammation causes neuronal injury and synapse injury, as well as favoring Aβ aggregation and plaque formation. Microglia is a key player in the initiation of neuroinflammation in AD brains [85]. The depletion of microglia results in a significant reduction in neuroinflammation and AD pathology in amyloidosis-based mouse models [86]. It is well acknowledged that the Trem2-dependent DAM has beneficial effects in suppressing pro-inflammatory signaling and fencing Aβ plaques [87,88]. In addition, scRNAseq and spatial transcriptomics analysis implicates the capability of Trem2-dependent DAM in phagocytic clearance of Aβ [55,72]. In this context, *Trem2* modulation may ameliorate AD pathology. However, attempts to overexpress *Trem2* in AD mouse models demonstrated a temporal effect. Jiang and colleagues introduced *Trem2* overexpression in APPswe/PS1dE9 (APP/PS1) mice through lentiviral delivery of mouse *TREM2* gene into the brain. The protective effects of *Trem2* overexpression against neuroinflammation, brain amyloidosis, and synapse loss were only observed at a young age before plaque formation [60]. However, *Trem2* overexpression lost its protection in old APP/PS1 mice demonstrating severe brain pathology [42]. A similar effect was observed in human TREM2 overexpression 5XFAD mouse model [41]. These findings seem to highlight a role of Trem2/TREM2 in preventing the development of AD-like pathology. The ineffectiveness of Trem2/TREM2 overexpression in aged mice could be explained by a floor effect, as Trem2 is highly expressed in the mouse models at the late stage of AD pathology. On the contrary to the effects of Trem2/TREM2 overexpression, it would be expected to see that Trem2 deficiency can exacerbate AD pathology. Unexpectedly, although Trem2 deficiency exacerbated amyloidopathy in old APP/PS1 mice, it reduced Aβ deposition in the young APP/PS1 mice brain [36]. Sheng and colleagues found decreased plaques but aggravated neuronal lesions in old Trem2-deficient PS2(N141I)/APPswe (PS2/APP) mice [89]. This raised a hypothesis that loss of Trem2 diminishes the capability of Trem2-dependent DAM in restraining Aβ plaques and results in enhancing soluble Aβ-induced neurotoxicity. Although many confounding factors such as the difference in mouse models and the potential impact of Trem2 on γ-secretase may puzzle the observations, these conflicting results briefly revealed the complexity of Trem2 functions. To this end, further investigation is needed for uncovering the impact of Trem2 signaling and Trem2-dependent DAM on amyloid pathology in AD.

## 4. Possible Dark Sides of Trem2

Despite the continually growing recognition of Trem2 in health and disease, the complexity of Trem2 in the pathogenesis of AD was noted. Although the TREM2 missense mutations are closely associated with increased susceptibility to the incidence of AD, TREM2 may not unconditionally show protection roles in AD pathology. In AD mouse models, the Trem2-dependent DAM markers are enriched in lysosome components (Figure 2B). However, the mature phagolysosome markers (e.g., Lamp1) were not upregulated in the DAM. Besides, the predominantly upregulated DAM marker, *Cst7*, encodes a potent inhibitor of lysosomal proteases [90,91]. Hence, the Trem2-dependent DAM may not form mature phagolysosome. In this context, we cannot exclude the possibility that Trem2-dependent DAM show less protection role in AD pathology. The gradually accumulation of DAM during AD pathological process may be a “primrose path” or an “expending in vain”.

Another issue is that the anti-inflammatory function of Trem2 signaling can induce negative effects. Recent studies on infection and cancer highlighted a role of Trem2 in the immune evasion of pathogens and cancer cells. Zhu and colleagues found that the infection of porcine reproductive and respiratory syndrome virus (PRRSV) induced Trem2 upregulation in porcine alveolar macrophage [92]. While the viral replication was promoted by *Trem2* overexpression and inhibited by the downregulation of *Trem2*. Such an effect is associated with the suppressive effect of Trem2 on PI3K/NF-κB signaling. In cancer, Trem2 was suggested to be a key regulator of myeloid suppressive cells [93]. Genetic depletion of Trem2 reduced regulatory myeloid cell phenotype and inhibited tumor growth [93,94]. Correlatedly, recent studies determined Aβ-induced microglial immune tolerance in 5XFAD mouse model [95]. While the application of interferon γ (IFNγ), a pro-inflammatory cytokine, attenuated microglial dysfunction and increased Aβ clearance. A reasonable explanation was that the excessive activation of Trem2 signaling participated in the immune tolerance, because it can suppress pro-inflammatory response and inhibit microglial functions of immune surveillance, phagocytosis, and lysosomal degradation.

To be more specific, we hypothesize a model in which Trem2 is beneficial at the early stage of AD when destructive inflammatory activations take place, and Aβ-induced microglial immune tolerance is insignificant. However, with the progression of amyloid pathology, the upregulation of Trem2 mitigates inflammatory response, promotes immune tolerance, and facilitates Aβ escape from microglial clearance. This hypothesis does not contradict the deleterious role of Trem2 deficiency in the development of AD, because Trem2 is critical for microglial physiology and loss of Trem2 results in insufficient microglial function. Future research to determine the influence of Trem2 on the development of immune tolerance in AD-related conditions will help to address the question. If the hypothesis is correct, upregulating TREM2 might help with the prevention of AD, while downregulating TREM2 in a sophisticated manner without affecting the physiological function of TREM2 could be a strategy for the treatment of AD.

## Figures and Tables

**Table 1:** Regulation and function of the major Trem2-dependent disease-associated microglia (DAM) markers.

Major DAM Markers	Putative or Verified Upstream Regulators	Putative or Verified Functions	Regulation in AD Mouse Models	Regulation in AD Postmortem Samples
Cst7	Trem2, Il10ra (inhibition), Tnf	Inhibiting proteases (papain and Ctsl) activity; possible immune regulation function	Upregulation in sorted microglia of PS2APP (GSE75431), 5XFAD (GSE65067), APP/PS1 (GSE74615), and Tau-P301S (GSE93180). Upregulation in bulk cortex of PS2APP (GSE75357).	Upregulation in bulk tissue of temporal cortex (GSE15222) and fusiform gyrus (GSE95587, GSE125583).
Trem2 (Stage 2)	Trem2, miRNA-34a (inhibition), LPS (inhibition), Tgfb1 (inhibition), Il4, Csf1, Csf2, PU.1	Receptor of amyloid-beta protein 42; immune signaling receptor for microglial activation, proliferation, migration, apoptosis, and expression of both pro-inflammation and anti-inflammation factors	Upregulation in sorted microglia of APP/PS1 (GSE74615). Upregulation in bulk cortex of PS2APP (GSE75357).	Upregulation in bulk tissue of temporal cortex (GSE15222) and fusiform gyrus (GSE95587, GSE125583).
Apoe (Stage 2)	Trem2, Hif1a (Csf2, Il5, Stat1, Tgfb1, Tnf), App (Ifng, Ngf, Tgfb1, Tnf), Map2k1 (Jak/Stat, Tgfb1, Chemokine, Il15), Stat1, Tgfb1, Tnf (inhibition)	Inhibiting apoptosis; accelerating chemotaxis; Il12 signaling and production in macrophage; quantity of neuroglia; lipid transport in CNS; innate and adaptive immune responses	Upregulation in sorted microglia of PS2APP (GSE75431), 5XFAD (GSE65067), APP/PS1 (GSE74615), and Tau-P301S (GSE93180).	Upregulation in sorted microglia (GSE125050), and purified microglial nucleus (snRNAseq, syn18485175).
Ctsd/Ctsb (Stage 1)	App (Ifng, Ngf, Tgfb1, Tnf), Ifng, Tgfb1, Tnf, Nfe2l2, Tp53	Lysosomal proteases; playing roles in APP processing or degradation	Upregulation in sorted microglia of APP/PS1 (GSE74615), and Tau-P301S (GSE93180). Upregulation in bulk cortex of PS2APP (GSE75357).	Upregulation in bulk tissue of temporal cortex (GSE15222).
Csf1	Hif1a (Csf2, Il5, Stat1, Tgfb1, Tnf), Egr1, Cebpa, Csf2 (Trem1), App (Ifng, Ngf, Tgfb1, Tnf), Csf1r, Il1b, Ifng, Tgfb1, Tnf	Inhibiting apoptosis; accelerating chemotaxis; tyrosine phosphorylation of protein; inhibition of cells; interaction of leukemia cell line; quantity of neuroglia; major cytokine for survival, proliferation and differentiation of myeloid cells	Upregulation in sorted microglia of PS2APP (GSE75431), 5XFAD (GSE65067), APP/PS1 (GSE74615), and Tau-P301S (GSE93180). Upregulation in bulk cortex of PS2APP (GSE75357).	Upregulation in bulk tissue of fusiform gyrus (GSE95587, GSE125583).
Lpl (Stage 2)	Cebpa, Ifng (inhibition), Nfe2l2, Tgfb1 (inhibition), Tnf (inhibition)	Inhibiting disorder of lipid metabolism; key enzyme in triglyceride metabolism	Upregulation in sorted microglia of PS2APP (GSE75431), 5XFAD (GSE65067), APP/PS1 (GSE74615), and Tau-P301S (GSE93180). Upregulation in bulk cortex of PS2APP (GSE75357).	Downregulation in bulk tissue of temporal cortex (GSE15222) and fusiform gyrus (GSE95587, GSE125583).
Spp1	Egr1, Cebpa, miRNA-135, IgE (Il8), Csf2, Zfp36 (Il8), Akt1 (Interferon, Jak/Stat, Trem1), Mavs, Pdx1, Igf1, Il1b, Il2, Tgfb1, Tnf, Ifng, PU.1	Accelerating chemotaxis; tyrosine phosphorylation of protein; interaction of leukemia cell line; cytokine for enhancing pro-inflammatory activation	Upregulation in sorted microglia of PS2APP (GSE75431), 5XFAD (GSE65067), APP/PS1 (GSE74615), and Tau-P301S (GSE93180). Upregulation in bulk cortex of PS2APP (GSE75357).	Upregulation in purified microglial nucleus (snRNAseq, syn18485175). Upregulation in bulk tissue of temporal cortex (GSE15222) and fusiform gyrus (GSE95587, GSE125583).
Cd9 (Stage 2)	Il5 (Cebpd, Il1), Cxcl12, Il2 (inhibition), Il5	Tyrosine phosphorylation of protein; chemotaxis of phagocytes; involved in platelet activation and aggregation	Upregulation in sorted microglia of APP/PS1 (GSE74615) and Tau-P301S (GSE93180). Upregulation in bulk cortex of PS2APP (GSE75357).	Upregulation in bulk tissue of fusiform gyrus (GSE95587, GSE125583).
Ccl6 (Stage 2)	Ifng, Tnf, Myc	Accelerating chemotaxis; promoting innate immune activation	Upregulation in sorted microglia of PS2APP (GSE75431), 5XFAD (GSE65067), APP/PS1 (GSE74615), and Tau-P301S (GSE93180). Upregulation in bulk cortex of PS2APP (GSE75357).	Not detected.
Itgax (Cd11c) (Stage 2)	Csf2, Ifng, Irf7, Stat1, Tgfb1 (inhibition), Tnf	Monocyte adhesion and chemotaxis	Upregulation in sorted microglia of PS2APP (GSE75431), 5XFAD (GSE65067), APP/PS1 (GSE74615), and Tau-P301S (GSE93180). Upregulation in bulk cortex of PS2APP (GSE75357).	Upregulation in bulk tissue of fusiform gyrus (GSE95587, GSE125583).
Tyrobp (Dap12) (Stage 1)	Trem2, Ifng (inhibition)	Quantity of neuroglia; a ligand binding by multiple receptors, e.g., Trem1 and Trem2	Upregulation in sorted microglia of PS2APP (GSE75431), 5XFAD (GSE65067), and APP/PS1 (GSE74615). Upregulation in bulk cortex of PS2APP (GSE75357).	Upregulation in bulk tissue of temporal cortex (GSE15222) and fusiform gyrus (GSE95587, GSE125583).
Igf1	Tp53, Estrogen, Tnf (inhibition)	Regulator of cellular proliferation; inducer of PI3K-AKT signaling pathway	Upregulation in sorted microglia of PS2APP (GSE75431), 5XFAD (GSE65067), APP/PS1 (GSE74615), and Tau-P301S (GSE93180). Upregulation in bulk cortex of PS2APP (GSE75357).	Downregulation in bulk tissue of temporal cortex (GSE15222) and fusiform gyrus (GSE95587, GSE125583).
Clec7a (Stage 2)	Il1, Csf2, Ifng (inhibition)	Tyrosine phosphorylation of protein; enhancer for pro-inflammatory activation	Upregulation in sorted microglia of PS2APP (GSE75431), 5XFAD (GSE65067), APP/PS1 (GSE74615), and Tau-P301S (GSE93180). Upregulation in bulk cortex of PS2APP (GSE75357).	Upregulation in bulk tissue of fusiform gyrus (GSE95587, GSE125583).
Axl (Stage 2)	Hif1a (Csf2, Il5, Stat1, Tgfb1, Tnf), App (Ifng, Ngf, Tgfb1, Tnf), Cxcl12, Stat1, Tgfb1	Inhibiting apoptosis; accelerating chemotaxis; Il15 production; chemotaxis of phagocytes	Upregulation in sorted microglia of PS2APP (GSE75431), 5XFAD (GSE65067), APP/PS1 (GSE74615), and Tau-P301S (GSE93180). Upregulation in bulk cortex of PS2APP (GSE75357).	Upregulation in bulk tissue of temporal cortex (GSE15222) and fusiform gyrus (GSE125583).
Cd63	Trem2	Cell surface receptor involved in activation of Akt, Fak/Ptk2, and Mapk; promoting cell survival, adhesion, and migration	Upregulation in sorted microglia of APP/PS1 (GSE74615), and Tau-P301S (GSE93180). Upregulation in bulk cortex of PS2APP (GSE75357).	Upregulation in bulk tissue of fusiform gyrus (GSE125583).
Ank	Hif1a, Tgfb1	Attaching integral membrane proteins to cytoskeletal elements	Upregulation in sorted microglia of PS2APP (GSE75431), 5XFAD (GSE65067), APP/PS1 (GSE74615), and Tau-P301S (GSE93180). Upregulation in bulk cortex of PS2APP (GSE75357).	Not changed.

Note: Putative or verified upstream regulators were acquired from QIAGEN IPA. Putative or verified functions were acquired from QIAGEN IPA and GeneCards [70]. Regulations in AD mouse models and in AD postmortem samples were acquired from The Myeloid Landscape 2. The (inhibition) in putative or verified upstream regulators indicated that the factor inhibits the expression of the DAM marker. Additionally, the upstream factors of a certain regulator were shown in an adjacent bracket. The annotation of genes or proteins were listed in “Supplementary File—Abbreviation and annotation”.

## Data Availability

Tabula Muris (https://tabula-muris.ds.czbiohub.org/); STRING (https://string-db.org/); The Myeloid Landscape 2 (http://research-pub.gene.com/BrainMyeloidLandscape/BrainMyeloidLandscape2/); scREAD (https://bmbls.bmi.osumc.edu/scread/); GeneCards (https://www.genecards.org/).

## Supplementary Materials

The following are available online at https://www.mdpi.com/2073-4409/10/2/321/s1, Materials and Methods, Transcriptomics data list, Abbreviation and annotation.
