# Supplementary material for: TREM2 Mediates Microglial Anti-Inflammatory Activations in Alzheimer’s Disease: Lessons Learned from Transcriptomics
Source: Cells. 2021 Feb 4;10(2):321. doi: 10.3390/cells10020321 (PMC7913972; doi:10.3390/cells10020321)
Supplement: Supplementary file 1 [file cells-10-00321-s001.pdf]

Supplementary File:

1. Materials and Methods

1.1 Metadata acquisition

The scRNAseq data of mouse *Trem2* expression was acquired from Tabula Muris (https://tabula-muris.ds.czbiohub.org/) [1]. The self-learning protein interaction network of mouse Trem2 was generated by STRING (https://string-db.org/) [2]. Meta-analysis on TREM2 / Trem2 regulations in human postmortem and multiple mouse models was acquired from The Myeloid Landscape 2 (http://research-pub.gene.com/BrainMyeloidLandscape/BrainMyeloidLandscape2/) [3]. The TREM2 / Trem2 regulations in microglial scRNAseq / snRNAseq data of individual human postmortem samples and individual mouse model samples were acquired from scREAD: A Single-cell RNA-Seq Database for Alzheimer's Disease (https://bmbls.bmi.osumc.edu/scread/) [4].

1.2 RNAseq and microarray data acquisition

Transcriptomics (RNAseq and microarray) raw data were downloaded from NCBI-GEO and NCBI-SRA. Briefly, the RNAseq reads data (GSE75431, GSE74615, GSE75357) were aligned to *Mus musculus* UCSC mm10 (RefSeq gene annotation) using STAR aligner Version 2.0.2 (Illumina Basespace) [5]. Differential gene expression (DEG) was performed by rlog Transformation function from DESeq2 software Version 1.1.0 (Illumina Basespace) [6]. Gene regulation was assessed by fold change calculation and significance was assessed with one-way ANOVA followed by Bonferroni's post hoc test. The significant DEGs (adj. P.Val<0.05) were selected for further bioinformatics analysis. The microarray data (GSE65067, GSE66926) were firstly analyzed using GEO2R tool of NCBI-GEO to generate DEGs. The significant up-regulations and down-regulations (adj. P.Val<0.05) were selected for bioinformatics analysis.

1.3 Bioinformatics analysis

Functional enrichment analysis was performed by FunRich3.1 according to the software protocol and default statistics setting [7]. Briefly, the significant DEGs lists were uploaded into software. Then the analysis of Gene Ontology-Biological Process pathway and the Gene Ontology-Cellular Component enrichment of the microglial subgroup markers was performed using the 'Gene enrichment - Analysis' function according to UniProt (https://www.uniprot.org/) protein database. Fold enrichment analysis of the Neurodegeneration-Related Modules against the LPS-Related Modules as background was performed using the 'Gene enrichment - Compare datasets - Fold quantity' function of FunRich3.1 to compare the major functional pathway differences between the 2 datasets. Mechanistic Network of Upstream Regulators, Regulator Effect Network, and Causal Network were performed by Ingenuity Pathways Analysis (IPA, QIAGEN) according to the software protocol and default statistics setting [8]. Putative upstream Cytokines, Transcriptional factors, and Receptors of the microglial subgroup markers were listed in table for illustration and comparison. The Ingenuity Pathways Analysis (IPA) software used in this publication was supported by the Biostatistics and Informatics Shared Resource, funded by the National Cancer Institute Cancer Center Support Grant P30 CA168524, and the Kansas IDeA Network of Biomedical Research Excellence Bioinformatics Core, supported in part by the National Institute of General Medical Science award P20GM103418.

2. Transcriptomics data list

RNAseq data:

GSE75431 (Srinivasan K, Friedman BA, Larson JL, Lauffer BE *et al.*, 2016, 'Sorted cells_PS2APP brains_7/13mo', NCBI-GEO, DOI: 10.1038/ncomms11295)

GSE74615 (Orre M, Kamphuis W, Osborn LM, Jansen AHP *et al.*, 2014, 'Acutely isolated murine cortical astrocytes and microglia: Alzheimer's disease vs wildtype', NCBI-GEO, DOI: 10.1016/j.neurobiolaging.2014.06.004)

GSE75357 (Srinivasan K, Friedman BA, Larson JL, Lauffer BE *et al.*, 2015, 'PS2APP whole tissue RNAseq', NCBI-GEO, DOI: 10.1038/ncomms11295)

Microarray data:

GSE65067 (Wang Y, Cella M, Mallinson K, Ulrich JD *et al.*, 2015, 'Expression data from WT and TREM2 deficient microglia in a mouse model of Alzheimer's disease', NCBI-GEO, DOI: 10.1016/j.cell.2017.07.023)

GSE66926 (Poliani PL, Wang Y, Fontana E, Robinette ML *et al.*, 2015, 'Expression data from WT and TREM2 deficient microglia in response to cuprizone mediated demyelination', NCBI-GEO, DOI: 10.1172/JCI77983)

3. Abbreviation and annotation

Trem2: Triggering Receptor Expressed on Myeloid Cells 2

Apoe: Apolipoprotein E

Cst7: Cystatin F

Il10ra: Interleukin 10 Receptor Subunit Alpha

Ctsl: Cathepsin L

Tnf: Tumor Necrosis Factor

miRNA34A: MicroRNA 34a

Tgfb1: Transforming Growth Factor Beta 1

Il4: Interleukin 4

Csf1: Colony Stimulating Factor 1

Csf2: Colony Stimulating Factor 2

PU.1: SPI1, Spi-1 Proto-Oncogene

Hif1a: Hypoxia Inducible Factor 1 Subunit Alpha

Il5: Interleukin 5

Stat1: Signal Transducer and Activator of Transcription 1

App: Amyloid Beta Precursor Protein

Ngf: Nerve Growth Factor

Map2k1: Mitogen-Activated Protein Kinase Kinase 1

Jak2: Janus Kinase 2

Il15: Interleukin 15

Ctsd: Cathepsin D

Ctsb: Cathepsin B

Nfe2l2: Nuclear Factor, Erythroid 2 Like 2

Tp53: Tumor Protein P53

Egr1: Early Growth Response 1

Cebpa: CCAAT Enhancer Binding Protein Alpha

Trem1: Triggering Receptor Expressed on Myeloid Cells 1

Csf1r: Colony Stimulating Factor 1 Receptor

Il1b: Interleukin 1 Beta

Lpl: Lipoprotein Lipase

Spp1: Secreted Phosphoprotein 1

miRNA-135: MicroRNA 135

Il8: CXCL8, C-X-C Motif Chemokine Ligand 8

Zfp36: ZFP36 Ring Finger Protein

Akt1: AKT Serine/Threonine Kinase 1

Mavs: Mitochondrial Antiviral Signaling Protein

Pdx1: Pancreatic and Duodenal Homeobox 1

Igf1: Insulin Like Growth Factor 1

Il2: Interleukin 2

Cd9: CD9 Molecule

Cxcl12: C-X-C Motif Chemokine Ligand 12

Ccl6: Chemokine (C-C motif) ligand 6

Myc: MYC Proto-Oncogene, BHLH Transcription Factor

Itgax: Integrin Subunit Alpha X

Irf7: Interferon Regulatory Factor 7

Tyrobp: Transmembrane Immune Signaling Adaptor TYROBP

Igf1: Insulin Like Growth Factor 1

Clec7a: C-Type Lectin Domain Containing 7A

Axl: AXL Receptor Tyrosine Kinase

Cd63: CD63 Molecule

Ank: Ankyrin 1

References:

1. Tabula Muris Consortium; Overall coordination; Logistical coordination; Organ collection and processing; Library preparation and sequencing; Computational data analysis; Cell type annotation; Writing group; Supplemental text writing group; Principal investigators. Single-cell transcriptomics of 20 mouse organs creates a Tabula Muris. *Nature* **2018**, *562*, 367–372, doi:10.1038/s41586-018-0590-4.
2. Szklarczyk, D.; Gable, A.L.; Lyon, D.; Junge, A.; Wyder, S.; Huerta-Cepas, J.; Simonovic, M.; Doncheva, N.T.; Morris, J.H.; Bork, P., et al. STRING v11: protein-protein association networks with increased coverage, supporting functional discovery in genome-wide experimental datasets. *Nucleic Acids Res* **2019**, *47*, D607–D613, doi:10.1093/nar/gky1131.
3. Srinivasan, K.; Friedman, B.A.; Etxeberria, A.; Huntley, M.A.; van der Brug, M.P.; Foreman, O.; Paw, J.S.; Modrusan, Z.; Beach, T.G.; Serrano, G.E., et al. Alzheimer's Patient Microglia Exhibit Enhanced Aging and Unique Transcriptional Activation. *Cell Rep* **2020**, *31*, 107843, doi:10.1016/j.celrep.2020.107843.
4. Jiang, J.; Wang, C.; Qi, R.; Fu, H.; Ma, Q. scREAD: A Single-Cell RNA-Seq Database for Alzheimer's Disease. *iScience* **2020**, *23*, 101769, doi:10.1016/j.isci.2020.101769.
5. Dobin, A.; Davis, C.A.; Schlesinger, F.; Drenkow, J.; Zaleski, C.; Jha, S.; Batut, P.; Chaisson, M.; Gingeras, T.R. STAR: ultrafast universal RNA-seq aligner. *Bioinformatics* **2013**, *29*, 15–21, doi:10.1093/bioinformatics/bts635.
6. Love, M.I.; Huber, W.; Anders, S. Moderated estimation of fold change and dispersion for RNA-seq data with DESeq2. *Genome Biol* **2014**, *15*, 550, doi:10.1186/s13059-014-0550-8.
7. Pathan, M.; Keerthikumar, S.; Ang, C.S.; Gangoda, L.; Quek, C.Y.; Williamson, N.A.; Mouradov, D.; Sieber, O.M.; Simpson, R.J.; Salim, A., et al. FunRich: An open access standalone functional enrichment and interaction network analysis tool. *Proteomics* **2015**, *15*, 2597–2601, doi:10.1002/pmic.201400515.
8. Kramer, A.; Green, J.; Pollard, J., Jr.; Tugendreich, S. Causal analysis approaches in Ingenuity Pathway Analysis. *Bioinformatics* **2014**, *30*, 523–530, doi:10.1093/bioinformatics/btt703.
